# Bronchial Stump Coverage Using a Free Pericardial Fat Pad to Prevent Fistula Formation: A Case Report

**DOI:** 10.7759/cureus.89870

**Published:** 2025-08-12

**Authors:** Sota Yoshimine, Toshiki Tanaka, Junichi Murakami, Naohiro Yamamoto, Kimikazu Hamano

**Affiliations:** 1 Department of Surgery and Clinical Science, Graduate School of Medicine, Yamaguchi University, Ube, JPN

**Keywords:** bronchial stump closure, bronchopleural fistula, complication, empyema, free pericardial fat, lung cancer

## Abstract

Bronchial stump fistulas develop into emergent and fatal conditions, so covering the bronchial stump with autologous tissues has been performed to prevent bronchial stump fistula. Covering the bronchial stump with a free pericardial fat pad is a technically simple and minimally invasive procedure. A 72-year-old man underwent left pneumonectomy for primary lung cancer with preoperative comorbidities associated with the risk of bronchopleural fistula, where coverage of the bronchial stump with a free pericardial fat pad was used. The histopathological diagnosis was small-cell carcinoma, and chemotherapy was initiated. Computed tomography performed 52 days postoperatively revealed a small amount of air in the fat pad, indicating bronchial stump dehiscence. The patient was asymptomatic and carefully continued chemotherapy. Five months later, empyema occurred, but fistula formation was avoided because the fat pad served as a separator. Coverage with a free pericardial fat pad can prevent fistula formation by separating the bronchial stump and thoracic space, even if bronchial stump dehiscence occurs.

## Introduction

Bronchial stump fistulas (BPF) develop into emergent and fatal conditions, such as aspiration pneumonia, respiratory failure, and bronchopulmonary artery fistulas. Covering the bronchial stump with autologous tissues, such as the intercostal muscle, thymus, pericardium, pericardial fat, and omentum, has been performed to prevent BPF [1]. Covering the bronchial stump with a free pericardial fat pad (FPFP) is a technically simple and minimally invasive procedure. Although there is no obvious evidence that FPFP coverage can prevent BPF, no BPFs occurred in 46 patients with FPFP coverage, and the fat remained on the bronchial stump when imaged with computed tomography (CT) five months after surgery [2]. In pigs, free fat tissue has been shown to remain in the thoracic cavity for a long time. Because of this, even if bronchial stump dehiscence cannot be prevented, the fat pad may separate bronchial and pulmonary arterial anastomoses [3]. In the case presented here, bronchial stump dehiscence and empyema occurred, but the FPFP prevented fistula formation between the bronchial stump and thoracic space.

## Case presentation

**Figure 1:**
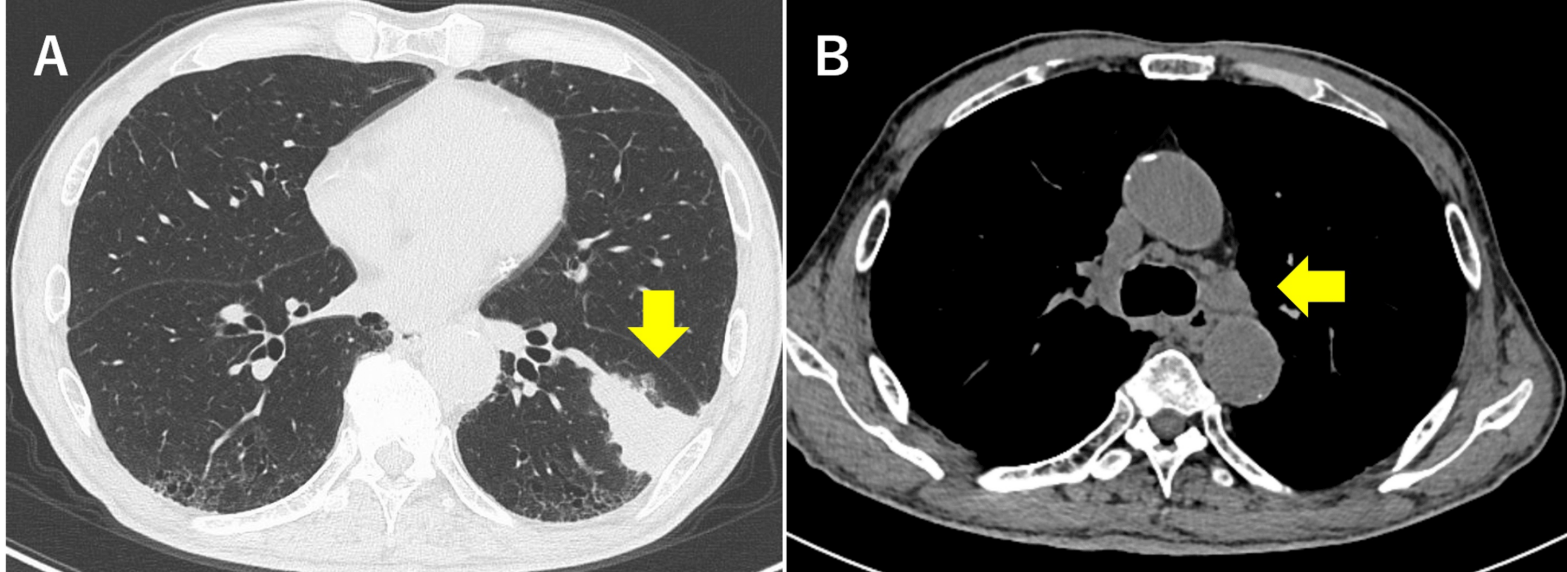
The tumor in the left lower lobe. (A) Computed tomography revealed a tumor (arrow) measuring 40×45 mm in the left lower lobe. (B) The #10 left (L), #11, and #12L lymph nodes were enlarged consecutively (arrow).

A 72-year-old man with several comorbidities (diabetes mellitus, severe renal dysfunction, and interstitial pneumonia) was referred to our hospital for lung cancer treatment. CT revealed a tumor measuring 40×45 mm in the left lower lobe of the lung (Figure 1, panel A), and the #10 left (L), #11, and #12L lymph nodes were enlarged consecutively (Figure 1, panel B). Furthermore, lymph nodes #4L and #5 were also enlarged. Due to severe renal dysfunction, contrast-enhanced CT was not performed, but positron emission tomography-CT showed 18F-fluorodeoxyglucose accumulation in these enlarged hilar and mediastinal lymph nodes (cT2bN2M0, stage IIIA).

**Figure 2:**
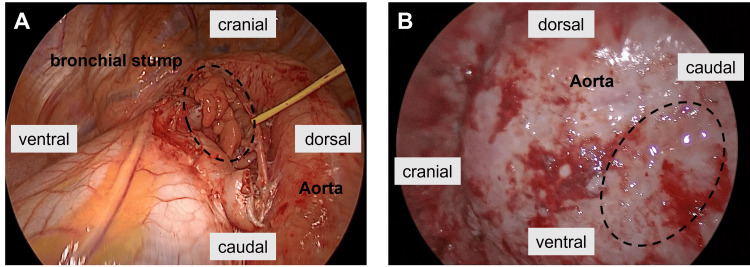
The bronchial stump covered with free pericardial fat. (A) First surgery. (B) Empyema without a fistula.

Bronchoscopic biopsy revealed malignant cells; however, the histological type, including small cell carcinoma or non-small cell carcinoma, could not be determined. Initial surgery was chosen after discussing with the multidisciplinary board because it was difficult to select an optimal chemotherapy regimen without a histological diagnosis, even in clinical IIIA-N2 (#4L, #5, #10L, #11, #12L). The surgery was performed via posterolateral thoracotomy. The hilar lymph nodes (#10L, #11, #12) were significantly enlarged and adhered to the pulmonary artery and bronchi. Although they could be sharply dissected away from the bronchi, dissection from the pulmonary artery was difficult. The pulmonary artery of the lingula segment was attached to lymph nodes at its bifurcation, making it difficult to preserve. It was determined that preserving the upper lobe by pulmonary artery reconstruction or sleeve resection would be difficult, so pneumonectomy was performed. The bronchial stump was covered with FPFP (Figure 2, panel A).

**Figure 3:**
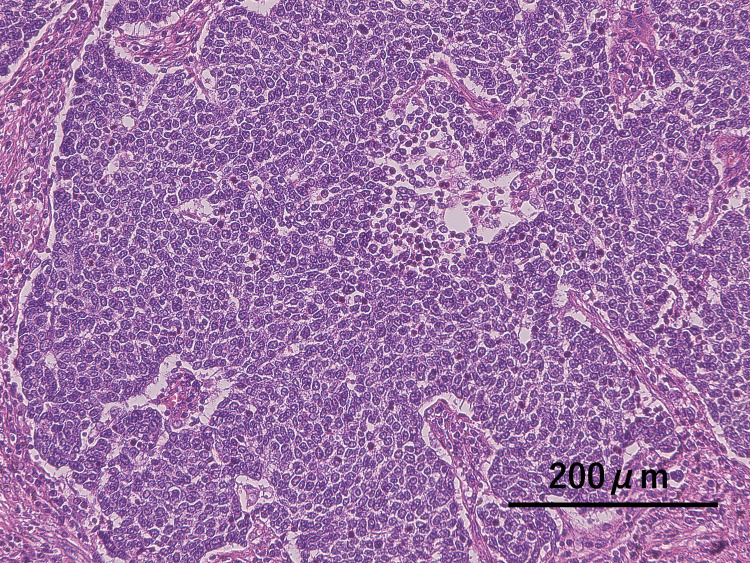
Histopathological image of tumor. The histopathological diagnosis was small-cell carcinoma. Small tumor cells with little cytoplasm proliferated densely (H&E stain).

**Figure 4:**
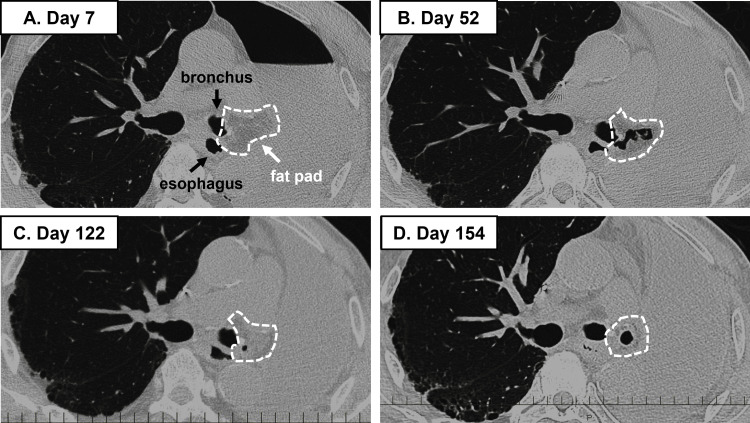
Air in the free pericardial fat covering the bronchial stump. (A) Day seven. (B) Day 52. (C) Day 122. (D) Day 154. Computed tomography performed 154 days postoperatively, at the onset of empyema, showed no evidence of aspiration pneumonia in the contralateral lung. The bronchial stump is outlined by uneven white dotted circles.

The postoperative course was uneventful. The histopathological diagnosis was small-cell carcinoma, and chemotherapy (carboplatin and etoposide) was initiated (Figure 3). However, CT on 52 days postoperatively revealed a small amount of air in the fat pad, indicating bronchial stump dehiscence (Figure 4, panel A).

Because the patient was asymptomatic and the air was limited to the fat pad (Figure 4, panels B, C), chemotherapy was continued with careful observation; however, 154 days postoperatively, empyema without a fistula occurred during the cytopenic phase in the fifth chemotherapy course. CT performed at the time of empyema did not reveal aspiration pneumonia in the contralateral lung (Figure 4, panel D). *Escherichia coli* was detected in the sputum and pleural fluid, and the infection could not be controlled with chest tube drainage or thoracoscopic debridement. Treatment with thoracic lavage and antibiotics was continued, but the infection was difficult to control, and open-window thoracostomy was performed. With careful observation during surgery, no penetration between the bronchial stump and the thoracic cavity was detected (Figure 2, panel B). Although the patient had no cancer recurrence, he died due to renal dysfunction nine months after the operation.

## Discussion

Covering the bronchial stump with various autologous tissues is performed to prevent BPF; however, no solid evidence for the efficacy of this procedure with any tissue exists, and which tissue to select is controversial [4]. Using an FPFP is simple and minimally invasive, and clinical images and animal experiments have shown that it remains in the thoracic cavity for a long time [2,3]. In our rat model experimental results, normal bronchial stumps were rarely reinforced, whereas covering the bronchial stump with free fat formed fibrous connective tissue rich in blood vessels around the stump, which reinforced the closure [5]. In the present case, covering the bronchial stump with FPFP resulted in bronchial stump dehiscence, but the fat pad served as a separator to prevent fistula formation. BPF immediately leads to serious infections and respiratory failure requiring emergency treatment. Here, the FPFP separated the bronchial stump from the thoracic cavity, allowing chemotherapy to continue and passively promoting tumor control. In another case similar to ours, the covered pedicled pericardial flap served as a separator and prevented air penetration between the dehiscent bronchial stump and the thoracic cavity, although it did not result in complete closure of the bronchial stump dehiscence [6]. It is important to note that when the pericardium is used, it is necessary to reconstruct the pericardial defect with an artificial material. In any case, FPFP use may be a valid option in situations where no reliable autologous tissue has been determined.

Since the asymptomatic and leaked air were limited to the fat pad, no additional treatment was administered; however, *Escherichia coli* was detected in the sputum and pleural fluid when empyema without a fistula occurred. To avoid the occurrence of empyema, preventive administration of antibiotics and additional procedures, such as bronchial embolization with fibrin glue may should be performed to prevent bacterial penetration during the asymptomatic period.

Covering the bronchial stump with an FPFP is performed primarily to prevent BPF; however, even if bronchial stump dehiscence occurs, the fat can act as a separator and prevent emergency and severe conditions, such as empyema with fistula or bronchopleural and pulmonary artery fistulas. Covering the bronchial stump with an FPFP could not prevent empyema due to dehiscence of the bronchial stump; however, it was able to prevent fistula formation.

## Conclusions

We presented a case of bronchial stump dehiscence and empyema following pneumonectomy, but the FPFP prevented fistula formation between the bronchial stump and the thoracic space. Coverage with an FPFP can prevent fistula formation by separating the bronchial stump and thoracic space, even if bronchial stump dehiscence occurs. In the absence of evidence-based prophylaxis for bronchopleural fistulas, it is reasonable to cover the bronchial stump with FPFP, a simple and minimally invasive method.
